# P73 and age-related diseases: is there any link with Parkinson Disease?

**DOI:** 10.18632/aging.100515

**Published:** 2012-12-18

**Authors:** Francesca Grespi, Gerry Melino

**Affiliations:** ^1^ Medical Research Council, Toxicology Unit, Leicester University, Leicester LE1 9HN, UK; ^2^ Biochemistry Laboratory, IDI-IRCCS, and University of Rome “Tor Vergata”, 00133 Rome, Italy

**Keywords:** P73, Parkinson disease, tyrosine hydroxylase, cell death, neurodegeneration

## Abstract

P73 is a member of the p53 transcription factors family with a prominent role in neurobiology, affecting brain development as well as controlling neuronal survival. Accordingly, p73 has been identified as key player in many age-related neurodegenerative diseases, such as Alzheimer's disease, neuroAIDS and Niemann-Pick type C disease. Here we investigate possible correlations of p73 with Parkinson disease. Tyrosine hydroxylase is a crucial player in Parkinson disease being the enzyme necessary for dopamine synthesis. In this work we show that levels of tyrosine hydroxylase can be influenced by p73. We also demonstrate that p73 can protect against tyrosine hydroxylase depletion in an *in vitro* model of Parkinson disease.

## INTRODUCTION

P73 is a transcription factors member of the p53 family [1, 2]. P73 gene contains two promoters that give rise to two main variant: one that retains the transactivation domain, TAp73 and a N-terminally truncated isoform, ΔNp73 [3, 4]. Even if the both of them retain a functional DNA-binding domain, they display opposing functions, with TAp73 being the pro-apoptotic isoform and ΔNp73 being the pro-survival one [5-9]. While p53 has been shown to play a prominent role in cancer and p63 in development, p73 has intermediate functions, including cancer [10-13], apoptosis [14-18], development [19-22], aging [23-26] and neurobiology [27-30]. In fact regarding this last trait, many works identifying p73 as a key player in neurobiology have been published, strongly supporting a role for this transcription factors in this field [31, 32]; it has been implied in Alzheimer's diseasedue to its effects on tau phosphorylation [25, 26, 33]. Furthermore, TAp73 is necessary for maintenance of neuronal precursors [34, 35], as well as in antagonizing proliferation when not necessary [36, 37]. Even the phonotypes of the animal models highlight the neuronal involvement of p73 [1]. More in details, the full p73 KO shows profound defects in brain development, displaying hippocampal dysgenesis and hydrocephalus [38], while TAp73−/− shows abnormal hippocampal anatomy [39] and DNp73 −/− is affected by severe reduction in neuronal density and present atrophic choroid plexuses [40, 41]. Recently it has also been published that TAp73 −/− mice show signs of impaired aging due to defects in mitochondrial respiration [42]; this was a striking discovery, since this feature has already been described for p53 but never before for p73 [43]. P73 has been involved in neuronal survival [44-48] as well as neuronal degenerative pathways such as the ones occurring in HIV-associated dementia [49] a syndrome that usually manifests at late stages of AIDS as a consequence of damaged central nervous system, but also more rarely of peripheral nerves [50-54]. P73 has been identified as a player in Niemann-Pick type C disease, a disorder that leads to accumulation of lipids both in the liver and central nervous system [55-58]. Furthermore p73 has also been implied in the most common form of dementia that is Alzheimer's disease [59-64]. TAp73α can induce tau phosphorylation, possibly implying a role of this particular variant in Alzheimer's disease [25, 33, 36, 65, 66]; this assumption is also supported by the fact that old p73+/− heterozygous mice display signs of Alzheimer's disease, such as reduced motor and cognitive function, accumulation of tau phosphorylation, tau kinase dysregulation and CNS atrophy [26].

Parkinson disease is a progressive degenerative disorder that presents loss of dopaminergic neurons in the substantia nigra [67-73] as well as failure in autophagic degradation of dysfunctional mitochondria [74-79] and misfolding of alpha-synuclein [80-82]. Many progresses, also thanks to the functional models developed, has been made for contrasting this pathology, however l-DOPA based treatment on long terms causes many sides effects as well as desensitization to drug response [67-70, 83, 84]. Many attempts have been made in order to find possible alternative treatments, such as use for example use of urocortin that was able to revert lesion-induced deficit in a rat PD model [85, 86]; another example is genipin that was able to protect N2a cells upon 6-OHDA induced cytotoxicity [87].

Here we investigate a possible involvement of p73 in this disease. Taking into account the prominent role that this transcription factor covers in brain development and degeneration, we investigated whether possible connections between p73 function and PD exists, focusing on influences on tyrosine hydroxylase levels, since this enzyme is necessary for dopamine synthesis [88-90].

## RESULTS

### Tyrosine hydroxylase promoter contains putative responsive elements for p73

**Figure 1:**
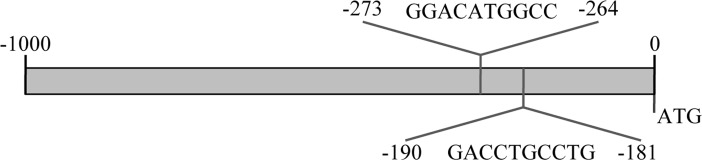
Th promoter encodes for putative p73 responsive elements Schematic representation of the p73 responsive elements in the promoter region of mouse tyrosine hydroxylase. Sequences with confidence of prediction ≥90% and indentified by all the predictive programs are reported, along with their sequence and position upstream than ATG start site.

We investigated the possibility that tyrosine hydroxylase (Th) could be a direct p73 transcriptional target. To this end we analyzed its promoter, screening for possible p73 responsive elements by using TFBIND (Transcription Factor Binding site) [91], TRED (Transcriptional Regulatory Element Database) [92, 93] and MatInspector (Genomatix) [94] and checked for congruency between the two prediction systems. In Figure 1, a schematic result of possible responsive elements identified by the programs is depicted.

### Tyrosine hydroxylase expression correlates with p73 levels

**Figure 2:**
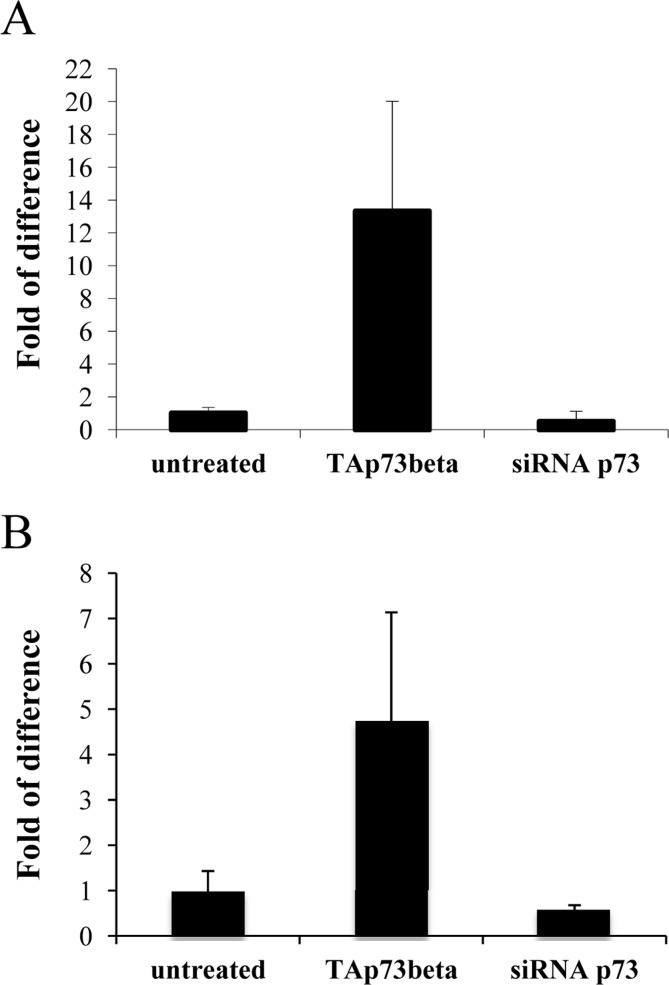
Tyrosine hydroxylase levels correlates with p73 CGN primary cells were transiently transfected (as indicated) and 48 hours later, collected and processed. Real-time PCR result of Th levels (**A**) and of p73 levels (**B**) are depicted. Experiment has been reproduced at least 3 times (data are represented as mean +/− SD).

Next, we wanted to check whether p73 could influence levels of Th. We used as initial system, primary cerebellar granule cells (CGN) that have been already used in *in vitro* models of Parkinson Disease (PD) [95, 96]. We transiently transfected these cells with a plasmid encoding for human TAp73β or siRNA for p73. We observed, by real-time PCR, that upon overexpression of TAp73β, levels of Th were increased of about 10 times. Moreover, knock down of p73 was leading to a reduction of around 50% in tyrosine hydroxylase levels (Figure 2A). We also confirmed transfection efficiency, even in this case by real-time PCR (Figure 2B).

### Tyrosine hydroxylase levels correlates with p73 transactivation potential

**Figure 3:**
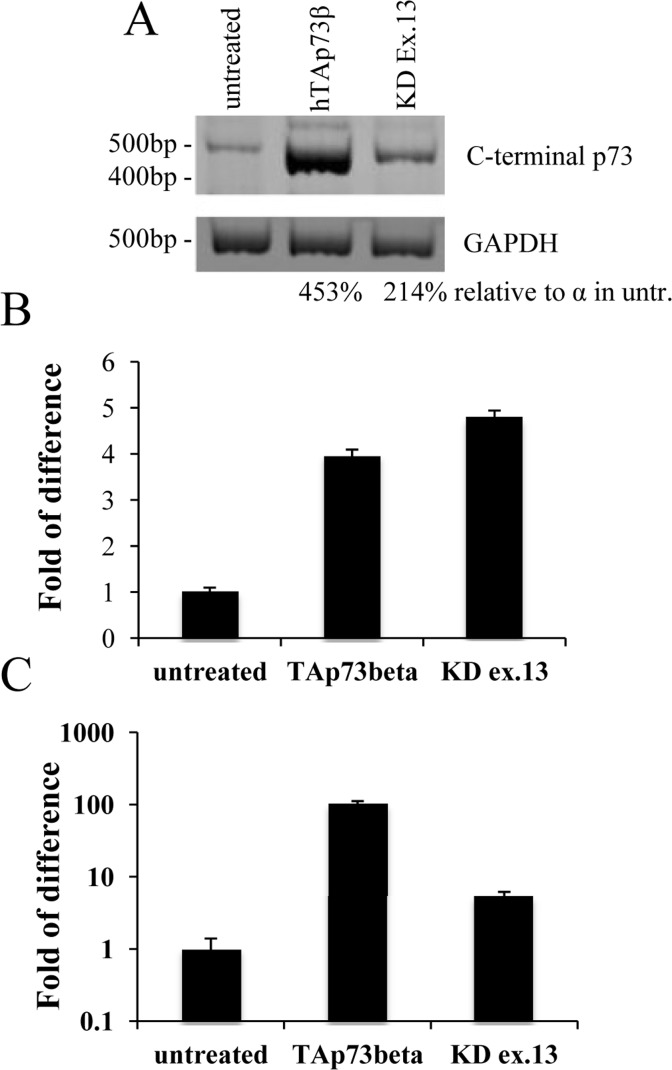
Tyrosine hydroxylase levels correlates with p73 transactivation potential N2a cells were transiently transfected with human TAp73β or shRNAs specific for exon 13; 48 hours later, cells were collected and processed. Semi-quantitative PCR showed that knock-down of exon 13 lead to a shift from α to β (**A**). Real-time PCR shows an increase of about 5 times in Th levels (**B**). In order to test efficiency of transfection, p73 levels were monitored (**C**). Experiment has been reproduced at least 3 times (data are represented as mean +/− SD). KD = knock-down, GAPDH = Glyceraldehyde 3-phosphate dehydro-genase, untr. = untreated. Experiment has been reproduced at least 3 times.

The p73α isoform is the only C-terminal variant that encodes for a fully functional Sterile Alfa Motif (SAM) [97-99], that has been identified as a repressor of transcription and apoptosis [100-102]. By interfering specifically with mouse p73 exon 13, it is possible to preclude the synthesis of a functional SAM domain [97]. We used a pool of 5 different shRNAs all specific for a portion of exon 13 and transfected N2a cells. Also N2a cells have been used already as an *in vitro* system for PD [103-106]. By semi-quantitative PCR (25 cycles), we noticed that KD of exon 13 was leading to a shift from α, that was the prominent isoform in untreated cells, versus β, with comparable levels as shown by densitometry analysis (Figure 3A). This shift, from a less to a more transactivating variant, lead to an increase on Th levels that were higher than the one found upon overexpression of human TAp73β (Figure B). TAp73 levels were monitored by qPCR as a read-out of transfection efficiency (Figure 3C).

### P73 counteracts depletion of Th by 6-OHDA

**Figure 4:**
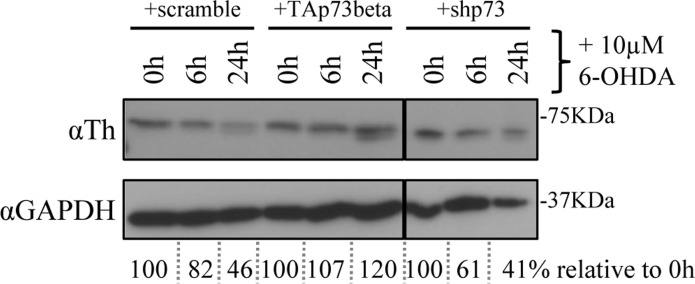
p73 counteracts depletion of Th by 6-OHDA N2a cells were transiently transfected with human TAp73 or shRNA against p73. After 48 hours, cells were treated with 10μM (final concentration) of 6-OHDA and collected at the indicated time points. Protein extracts were subjected to western blot analysis and quantified with densitometry. 6-OHDA = 6-hydroxydopamine, Th = tyrosine hydroxylase, GAPDH = Glyceraldehyde 3-phosphate dehydrogenase. Experiment has been reproduced at least 3 times.

N2a cells were transiently transfected with TAp73 or siRNA for total p73; 48 hours later, cells were treated with 10μM of 6-hydroxydopamine (6-OHDA) as an *in vitro* model for Parkinson Disease [107, 108]. Cells were collected at the indicated time points and levels of Th were monitored by western blot analysis. Overexpression of p73 was sufficient to avoid Th downregulation upon incubation with 6-OHDA. On the other hand, knock down of p73 was accelerating this process, as underlined also by densitometry analysis (Figure 4).

## DISCUSSION

We identified by screening the promoter region of tyrosine hydroxylase a possible responsive element of p73 (Figure 1). This has been confirmed in three distinct predictive databases: two responsive elements, with a confidence of prediction higher than 90%, suggests that Th is a potential target of p73. In line with these findings, in CGN primary cells there was an induction of mouse Th of about 15 times, upon overexpression of human TAp73β (Figure 2). This is an indication of how strong p73 can induce tyrosine hydroxylase, since this increase was resulting upon overexpression of a human p73 variant, while the upregulation that we monitored was the one of mouse endogenous Th. Further proof of this was that silencing of mouse total p73 was causing a decrease to a comparable extent in Th levels, strongly supporting the hypothesis of tight co-regulation between p73 and Th. Another importance aspect of p73 is that its different isoforms have different transactivation potential [101, 109, 110]. The TAp73α variant has a lower transactivation potential than the β isoform [101, 102, 109] that lacks exon 13, leading to a loss of functionality of the SAM domain [97, 99, 111]. Since specific KD of exon 13 lead to a shift from α to β (Figure 3A), we exploited this fact to monitor levels of Th driven by the β isoform in a more physiological context. Even if upon KD of exon 13, β levels were less than half than the overexpression of the human variants, we highlighted an increased of five versus four times in N2a cells respectively. This result further indicates that p73 might affect PD, since physiological levels of p73β were potent inducer of Th. A similar outcome was found in two different *in vitro* systems. The fold of induction of Th in CGN was greater than N2a; this could be related to the fact that CGN are dopaminergic cells [112-114] while N2a are not [104, 115]. Another intriguing result was the outcome of the *in vitro* PD induction with 6-OHDA. Indeed, TAp73β has a protective role in shielding cells against Th decrease, that is one of the main steps for the development of Parkinson Disease [116, 117]. We don't know whether ΔNp73 could play a role in this scenario, but it would be really interesting to investigate also on this matter, since also ΔNp73 has been reported to play an important role in brain development and function, but also in aging [47, 118, 119]. Furthermore, ΔNp73 plays a critical role in maintenance of developmental as well as adult neurons [118-120]. Following this line, it would be important to study specific p73 isoforms role, also focusing on the C-terminal variants of p73, that have not been fully characterized yet.

In conclusion, here we reported the ability for p73 to regulate tyrosine hydroxylase and by doing this, protecting against events that can lead to Parkinson disease.

## MATERIALS AND METHODS

### Cells cultures and substances

Cells were cultured at 37 °C in 5% CO_2_ in culture medium. N2a were purchased from ATCC (#CCL-131) and maintained in a mix of 45% DMEM high glucose, 45% Optimem and 10% fetal bovine serum, 250 mM L-glutamine, 1U/ml penicillin/streptomycin (all Gibco). Cerebellar Granule Cells were derived from cerebellum of P7 C57Bl/6 mice and generated as already published [95]. Mice were bred and subjected to listed procedures under the project license released from the United Kingdom Home Office. 6-OHDA was purchased from Sigma-Aldrich.

### Transfection

Transfections were carried out by Lipofectamine 2000 reagent (Invitrogen) according to the manufacturer's instructions. Cells were transfected with human TAp73β (GeneScript) or siRNA for p73 (Accell siRNA Dharmacon), or five shRNAs pool specific for exon 13 (Genecopoeia). After 48 hours, cells were harvested for protein and RNA extraction. Each experiment was performed at least in triplicate.

### RNA Extraction and qRT-PCR

RNA was extracted using TRIzol (Invitrogen) and following manufacturers guidelines. After extraction, RNA was quantified with NanoDrop 2000 (ThermoScientific) and 5μg were treated with DNase I (Sigma) in order to eliminate DNA contamination. cDNA was reversed transcribed using RevertAid H Minus First Strand cDNA synthesys kit (Fermentas). qRT-PCR was performed in an ABI PRISM 7000 Sequence Detection System (Applied Biosystem) with SYBR green ready mix (Applied Biosystem) and specific primers (please see primers session). Actin or 18S gene was used as internal control. Gene expression was defined from the threshold cycle (Ct), and relative expression levels were calculated by using the 2^−ΔΔCt^ method after normalization with reference to expression of housekeeping gene (GAPDH). Semi-quantitative PCR was performed using GoTaq DNA Polymerase (Promega) and the following cycle conditions: 5 min at 95°C; 30 s at 95°C, 1min at 58°C, 1 min at 72°C 30 cycles and 10 min at 72°C. PCR product was run on a 10% acrylamide gel (BioRad) and stained afterwards for 10 min in a 0.5μg/ml ethidium bromide (Sigma-Aldrich) solution.

### Western Blotting

Proteins were extracted with RIPA buffer containing cocktail inhibitors (Roche) and concentration was determined using a Bradford dye-based assay (Biorad). Total protein (50 μg) was subjected to SDS-PAGE followed by immunoblotting with appropriate antibodies at the recommended dilutions. The blots were then incubated with peroxidase linked secondary antibodies followed by enhanced-chemiluminescent detection using Super Signal chemiluminescence kit (Thermo scientific). Antibodies: rabbit polyclonal anti tyrosine hydroxilase (1:1000; Calbiochem), mouse monoclonal anti GAPDH (1:10000; Sigma-Aldrich). Densitometry analysis was achieved by using ImageJ software.

### Primers

Real-time PCR:

mTAp73 FWD 5'-GCACCTACTTTGACCTCCCC-3'
mTAp73 REV 5'-GCACTGCTGAGCAAATTGAAC-3'
mTh FWD 5'-CTTTGACCCAGACACAGCAG-3'
mTh FWD 5'-ACAAGCTCAGGAACTATGCC-3'
actin FWD 5'-GGCTGTATTCCCCTCCATCG-3'
actin REV 5'-CCAGTTGGTAACAATGCCATGT-3'
18S FWD 5'-AGTTCCAGCACATTTTGCGAG-3'
18S REV 5'-TCATCCTCCGTGAGTTCTCCA-3'

Semi-quantitative PCR:

mp73-X10 FWD: 5'-gagatcttgatgaaagtCAA gg-3'
mp73-X14 REV: 5'-GCATTTCCGTGTGCGCCAC-3'
GAPDH FWD 5'-CAAGGTCATCCATGACAACTTG-3'
GAPDH REV 5'-GTCCACCACCCTGTTGCTGTAG-3'

## Abbreviations

Th: tyrosine hydroxylase
TA: TAp73
ΔN: ΔNp73
6-OHDA: 6-hydroxydopamine
PD: Parkinson disease
